# Jejunal Perforation: A Rare Presentation of Burkitt's Lymphoma—Successful Management

**DOI:** 10.1155/2014/538359

**Published:** 2014-06-04

**Authors:** Samir Ranjan Nayak, Ganni Bhaskara Rao, Subramanya Sarma Yerraguntla, Sisir Bodepudi

**Affiliations:** ^1^Department of Surgery, GSL Medical College & General Hospital, NH-5, Lakshmipuram, Rajahmundry 533294, India; ^2^Department of Medical Oncology, GSL Medical College & General Hospital, Rajahmundry 533294, India

## Abstract

Malignant tumors of the small bowel presenting as acute abdomen are a rare occurrence. Burkitt's lymphoma presenting as a surgical emergency needing emergency laparotomy is an uncommon presentation of this tumor. We present an interesting case of jejunal perforation as a first manifestation of Burkitt's lymphoma which was successfully managed with surgical resection, high dose chemotherapy, and good supportive care.

## 1. Case Report

### 1.1. Initial Presentation

**Figure 1:**
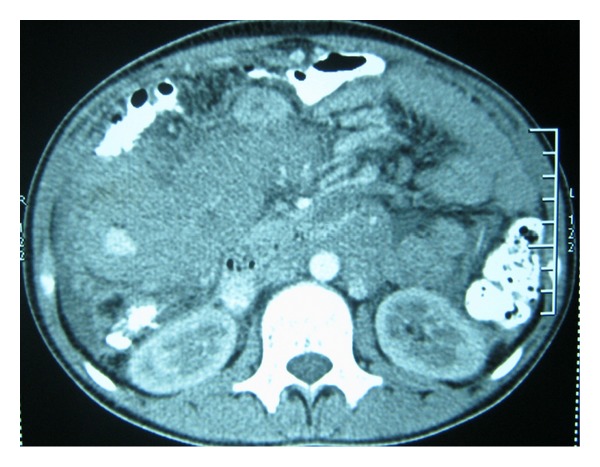
CECT abdomen-bowel mass at the right lumbar intussusceptions.

17-year student presented to the emergency ward with pain in the upper abdomen of 7 days duration, loose motion and occasional bilious vomiting. He had no significant past medical history. On general examination, there was gross pallor, temperature of-100-degree farenheight and a pulse rate of 107/minute. There was mild abdominal distension with a visible mass over the right hypochondrium. A tender mass of 4 × 5 cm^2^ was palpable over right hypochondrium with localized rigidity. Bowel sound was sluggish. His hemoglobin level was 4 gm/dL with a leukocyte count of 11 × 10^9^/L. The retroviral status was negative. The clinical diagnosis was made as small bowel intussusceptions with the differential diagnosis of appendicular mass, intestinal tuberculosis with clumped bowel, there were no air fluid levels or air under the diaphragm on an erect abdominal X-ray. The US abdomen and CECT abdomen report favored bowel intussusceptions with minimal fluid in the pelvis (Figure 1).

### 1.2. Surgical Course

**Figure 2:**
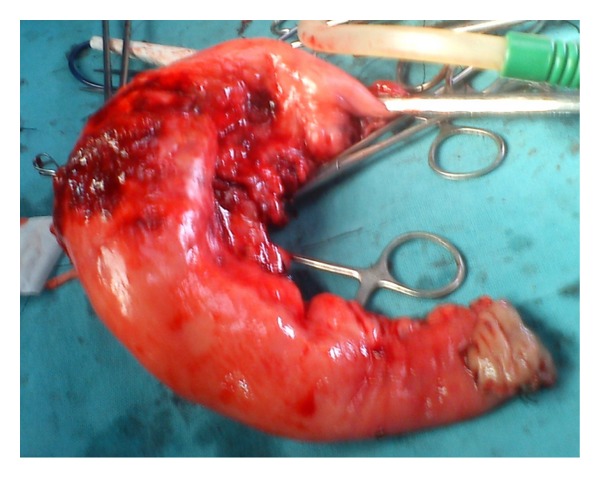
On laparotomy thickened jejunum with a small perforation at antimesenteric border.

**Figure 3:**
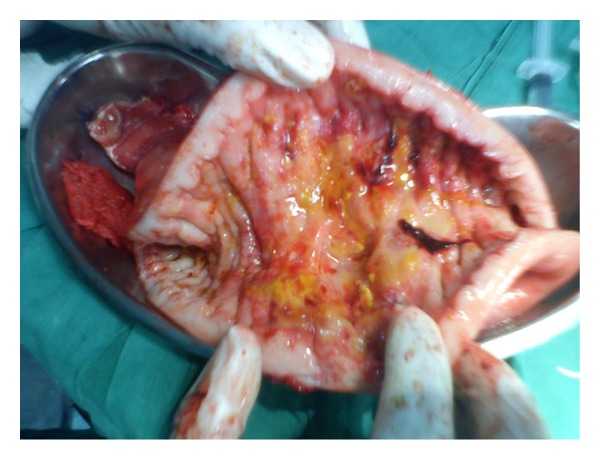
Cut-open jejunum;* fish flesh* appearance.

The patient underwent emergency laparatomy. There were purulent collections in the right paracolic gutter and pelvis. The proximal jejunum at 10 cm was grossly thickened with perforation of 0.7 cm at the antimesenteric border. There were multiple large nodes in the mesentry of the thickened jejunum (Figure 2). Resection and end-to-end anastomosis was done. Cut section of jejunum had a* fish flesh* appearance (Figure 3).

#### 1.2.1. Post-Operative Status

**Figure 4:**
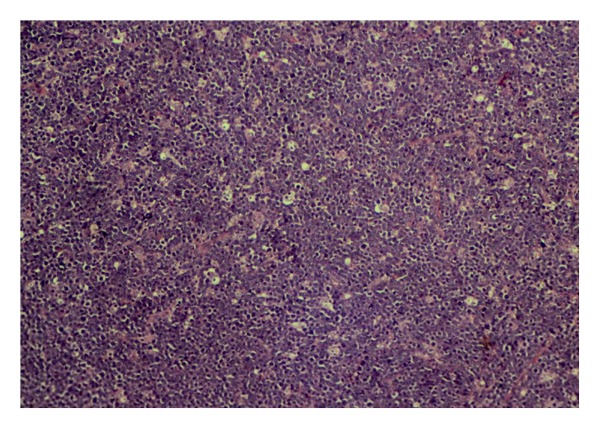
Microscopy-tumor cells arranged in diffuse sheets admixed with numerous macrophages giving a “starry sky” pattern (H&E, x100).

Post operatively the patient needed ventilator support with parental nutrition. The microscopic sections from the resected jejunum, and the lymph nodes showed the tumor cells arranged in diffuse sheets admixed with numerous macrophages giving a “starry sky” pattern (Figure 4). The histoptahological diagnosis was Non-Hodgkin lymphoma of jejunum-likely to be Burkitt's type. The surgical margin was free from tumor. The tumor stained positive for LCA- and PAN-B cell. CD 10, CD 20, BCL-6 was positive and KI-67 found positive in more than 80% of the cells. The peripheral smear did not show any blast cells. The bone marrow aspiration revealed hyperplastic marrow. Serum LDH level was 1361.5 U/L. The final postoperative diagnosis was Burkitt's Lymphoma of jejunum, Ann Arbor Stage II AEX, IPI Score 3: High Intermediate Risk, Lugano Stage: II E Risk Stratification For Treatment: High Risk [1].

The patient was started chemotherapy from 7th postop day. As a high-risk patient he received 4 total cycles (2 cycles of CODOX-M alternating with 2 cycles of IVAC). The chemotherapy scheduled was Inj cyclophosphamide 800 mg/m^2^ IV on day 1, and 200 mg/m^2^ IV on days 2–5. Inj Adriamycin 40 mg/m^2^ IV on day 1, Vincristine 1.5 mg/m^2^ IV on days 1 and 8 (cycle 1), as well as on days 1, 8, and 15 (cycle 3). Inj Methotrexate 1200 mg/m^2^ IV over 1 hour on day 10 and then 240 mg/m^2^/h for the next 23 hours; leucovorin rescue begins 36 hours from the start of the methotrexate infusion. Intrathecal cytarabine 70 mg on days 1 and 3. Intrathecal methotrexate 12 mg on day 15. IVAC (cycles 2 and 4) Inj Ifosfamide 1500 mg/m^2^ IV on days 1–5, with mesna. Inj Etoposide 60 mg/m^2^ IV on days 1–5. Inj Ara C 2 g/m^2^ IV every 12 hours on days 1-2. Inj GCF 300 Microgram sc daily 24 hous after completion of chemotherapy. Intrathecal methotrexate 12 mg on day 5.

Marked improvement was noticed after the initiation of chemotherapy. Abdominal distension decreased and the patient tolerated oral fluids. He had vincristine induced peripheral neuropathy involving right lower limb and paralytic ileus following second CODOX-M course which was managed conservatively with symptomatic treatment, component therapy with packed RBC and platelet support. He developed Grade 4 neutropenia with fever which was managed with broad-spectrum antibiotics and colony stimulating factors.

After completion of chemotherapy, abdomen was soft, serum LDH was normal and CECT abdomen showed no residual disease. Patient was advised follow up once in a 3 month for 2 years and then once in 6 month. Patient was last followed up in Dec 2013 and had no evidence of disease clinically or on investigations.

## 2. Discussion

Burkitt's lymphoma (BL) is a high-grade aggressive B cell Non Hodgkin's lymphoma. It is one of the fastest growing human malignancies, with a 100% replication rate and a doubling time of approximately 18–25 hours [2]. The rapidity of volumetric doubling of this neoplasm may lead to an acute abdominal presentation, which may mimic other less rare diseases.

Lymphoma constitutes 15%–20% of all small intestine neoplasms. The Ileum is the most common site (60%–65%) involving small intestine lymphoma followed by jejunum (20%–25%), duodenum (6%–8%) and other sites (8%-9%).

The clinical presentation of small intestinal lymphoma is nonspecific and the patient may have symptoms, such as colicky abdominal pain, nausea, vomiting, distension. Rarely patient may present with acute intestinal obstruction or gastrointestinal bleeding. The intestinal obstruction resulted from direct compression of the lumen by an expanding mass or by intussusceptions of the projecting intraluminal mass. [3–5]. The case presented with all the features of intussusceptions in form of pain abdomen, and vague abdominal lump at right hypochondium.

Abdominal sonogram/CECT may show thickening of a segment of bowel or a target/pseudo kidney sign representing intussusceptions [6]. To diagnose primary small intestinal tumors clinically is difficult and in the emergency setting, the diagnosis of lymphoma is usually made postoperatively.

The purulent fluid collection in the abdominal cavity is due to perforation of proximal obstructed bowel. Spontaneous intestinal perforation is an uncommon complication of BL, even though previously reported in children [7]. Possible explanations for spontaneous gastrointestinal perforation are tumor necrosis, immune suppression and protein malnutrition.

Preoperative diagnosis is difficult in Burkitt's lymphoma involving the small bowel; therefore, the role of surgery is limited to the management of complications like perforation or obstruction. In the present case laparatomy was carried out for intussception with features of peritonitis [8, 9].

Aggressive early chemotherapy must be initiated even if resection of bowel and complete resection of the mass has been performed as there have been no reports of cure with the resection alone.

The histopathological types seen in intestinal lymphoma of children and adolescents include Burkits Lymphoma, Diffuse Large B Cell Lymphoma (DLBCL) and rarely Hodgkin's Lymphoma [1, 7]. and hence immune histochemistry is mandatory to differentiate the type of lymphoma.

The typical immunophenotype of BL is sIg+, CD10+, CD19+, CD20+, CD22+ TdT−, Ki67+ (>95%), BCL2−, BCL6+, and simple karyotype with MYC rearrangement. Most cases (80%) of classical BL are characterized by t(8; 14) which results in the juxtaposition of MYC gene from chromosome 8 with the IgH region on chromosome 14. Other variants with MYC rearrangements [t(8; 22) or t(2; 8)] are less common [1]. In the presented case the tumor stained positive for LCA- and PAN-B cell. CD 10, CD 20, BCL-6 was positive and Ki-67 expression was found in more than 80% of the cells.

The recommended workup in a case of intestinal lymphoma includes complete physical examination with attention to node-bearing areas, including waldeyer's ring, and to size of liver and spleen. The performance status, serum LDH, comprehensive metabolic panel, serum uric acid, chest/abdominal/pelvic CT with contrast, lumbar puncture, flow cytometry of cerebrospinal fluid, and bone marrow aspiration should taken into account [1]. For the presented case the serum LDH was grossly elevated with hypercellular bone marrow and normal CSF analysis. There was no hepatosplenomegaly.

Risk stratification categories include Low Risk (normal LDH, completely resected abdominal lesion or single extra-abdominal mass <10 cm) and High Risk. High risk includes patients presenting with intestinal obstruction or perforation. High Risk patients are given CALGB 10002 regimen + rituximab or CODOX-M (original or modified) ± rituximab or Dose-adjusted EPOCH or HyperCVAD + rituximab [1, 7]. In a prospective study, low-intensity EPOCH-R-based treatment was highly effective in adults with sporadic or immunodeficiency-associated Burkitt's lymphoma [10]. There was a trend in favor of superiority of rituximab-containing therapy in all efficacy end points, including 3-year Progress free survival PFS (74% versus 61%) and 3-year OS (77% versus 66%). In addition, fewer relapses were observed [11].

Dose modifications are required depending upon pre chemo hematological and biochemical parameters. Dose-adjusted EPOCH can be given for high-risk patients not able to tolerate aggressive treatment. Taking the patient age, pre-op clinical parameters, postop status and economic status into consideration the patient was started on CODOX-M/IVAC regimen in alternate cycles for four courses.

BL is curable in a significant subset of patients when treated with dose-intensive, multiagent chemotherapy regimens that also incorporates CNS prophylaxis. About 60% to 90% of pediatric and young adult patients with BL achieve durable remission if treated appropriately. The 5 year survival estimate is 56% and durable remission may be possible in approximately 60% of patients with BL [1].

In general, patients with low-stage disease (i.e., single extra-abdominal/extrathoracic tumor or totally resected intra-abdominal tumor) have an excellent prognosis (a 5-year survival rate of approximately 90%), Patients with high-stage (stage III or stage IV) mature B-lineage non-Hodgkin lymphoma (NHL) (Burkitt or Burkitt-like lymphoma a and diffuse large B-cell lymphoma) have an 80% to 90% long-term survival [1]. On 18 month, follow up the patient had no clinical symptoms and no radiological relapse.

The Event free survival has been markedly improved with multi-agent intensive chemotherapy like CODX-M/IVAC. In the presented case after establishing the diagnosis on seventh postop day the chemotherapy was started with a marked improvement [1, 7].

## 3. Conclusion

Burkitt's lymphoma is an aggressive, rapidly progressive malignancy and may present as jejunal perforation. The successful outcome depends on early postop diagnosis and aggressive chemotherapy with supportive care.
